# Shared immune-inflammatory mechanisms between ulcerative colitis and periodontitis: a multi-omics analysis

**DOI:** 10.3389/fimmu.2025.1668277

**Published:** 2025-08-27

**Authors:** Hongjiao Li, Peimin Li, Xiaofeng Guo, Yan Zhou, Shaowei Ma, Xin Gao

**Affiliations:** ^1^ The Stomatology Department of Shanxi Provincial People Hospital, Taiyuan, Shanxi, China; ^2^ The Gastroenterology Department of Shanxi Provincial People’s Hospital, Taiyuan, Shanxi, China; ^3^ Department of Gastrointestinal Disease Center, The First Hospital of Hebei Medical University, Shijiazhuang, Hebei, China; ^4^ Department of Gastrointestinal Surgery, The Second Hospital of Hebei Medical University, Shijiazhuang, Hebei, China

**Keywords:** ulcerative colitis, periodontitis, CXCL6, oral-gut axis, Mendelian randomization, immune infiltration

## Abstract

**Background:**

Ulcerative colitis (UC) and periodontitis (PD) are chronic inflammatory diseases with increasing evidence of bidirectional communication through the oral–gut axis. However, the immunological mechanisms underlying their co-occurrence remain largely unclear.

**Methods:**

We conducted a bidirectional Mendelian randomization (MR) analysis to evaluate potential causal relationships between UC and PD. Transcriptomic data from public repositories were integrated to identify shared differentially expressed genes. Immune-related genes were further screened using three machine learning approaches. Enrichment analysis and immune cell infiltration profiling were performed to explore underlying mechanisms. A rat model combining UC and PD was established to validate key findings *in vivo*.

**Results:**

MR analysis revealed a unidirectional causal effect of UC on PD. Among the intersected immune-related genes, CXCL6 was identified as a hub gene significantly upregulated in both UC and PD. It was associated with neutrophil infiltration and pathways related to chemokine signaling and mucosal barrier disruption. In a dual-disease rat model, CXCL6 expression was further elevated in colonic tissues compared to UC alone, aligning with aggravated epithelial damage.

**Conclusion:**

Our study identifies a shared immune signature between UC and PD, highlighting CXCL6 as a pivotal mediator. These insights deepen understanding of oral–gut mucosal interactions and inform future biomarker and mechanistic studies.

## 1 Introduction

Ulcerative colitis (UC) is a chronic gastrointestinal disorder marked by abnormal activation of the mucosal immune system within the intestines (1–3). This immune response presents as symptoms including abdominal pain, diarrhea, and bloody stools (4). Approximately 15% to 40% of patients with UC present with extraintestinal manifestations (EIM), which include oral lesions, hepatobiliary dysfunction, arthralgia, cutaneous symptoms, and neurological complications. The pathogenesis of EIM remains incompletely understood; it may represent an extension of the intestinal immune response or occur independently of intestinal inflammatory processes (5, 6).

Periodontitis (PD) is a prevalent inflammatory condition affecting the supporting structures of teeth (7). This immunoreactive disease, primarily driven by bacterial infection, is characterized by gingival inflammation, the formation of periodontal pockets, alveolar bone resorption, and potential tooth mobility. The condition arises mainly from bacterial accumulation in the oral cavity, triggering an inflammatory response that damages the gums and adjacent tissues. If left untreated, periodontitis can have significant repercussions on both physical and mental well-being.

The relationship between UC and PD remains complex and controversial. Schmidt et al. (8) observed that patients with UC exhibit more severe periodontal disease. Meanwhile, the prevalence of UC is elevated in individuals with periodontitis (9–11). Interestingly, a large cohort study involving over 20,000 participants found that periodontitis was associated with a reduced risk of inflammatory bowel diseases (IBD) (12). To further investigate the causal relationship between UC and PD, several studies have utilized bidirectional Mendelian randomization (MR) analysis. For example, Qing et al. (13) identified periodontitis as a potential risk factor for UC (odds ratio [OR], 1.13; 95% confidence interval [CI], 1.01–1.26; P = 0.027), whereas UC did not appear to exacerbate periodontal disease. On the other hand, Wang et al. (14) found an association between UC and periodontitis (OR, 1.074; 95% CI, 1.029–1.122; P = 0.001), but concluded that periodontitis was not linked to the development or worsening of UC. These findings highlight the complexity of the UC-PD relationship, with varying conclusions regarding directionality and causality.

The relationship between UC and PD is significantly mediated by the oral-gut axis (15). Pathogens associated with periodontal disease can migrate from the oral cavity to the gut, exacerbating intestinal inflammation through several pathways, including disruption of the gut microbiome and barrier, as well as the release of inflammatory factors that trigger immune responses (16–18). Concurrently, localized immune reactions to intestinal dysbiosis can initiate systemic T-cell-mediated responses and cytokine release, potentially leading to the development of oral lesions (19, 20). Thus, investigating the shared immune mechanisms may represent a pivotal approach to addressing the co-occurrence of ulcerative colitis and periodontitis.

In this study, we began by investigating the potential relationship between UC and PD using Mendelian randomization analysis. Then, we synthesized data from multiple GEO databases and identified that the differentially expressed genes common to UC and PD are significantly linked to various immune pathways. Our analysis culminated in the identification of CXCL6 as a pivotal immune hub gene that connects both conditions, determined through advanced machine learning techniques. To further substantiate our findings, we constructed a dual disease model in rats, which allowed us to validate the expression levels of CXCL6. This work offers novel insights into the molecular immune mechanisms that underlie the association between UC and PD.

## 2 Materials and methods

### 2.1 Mendelian randomization analysis

**Figure 1:**
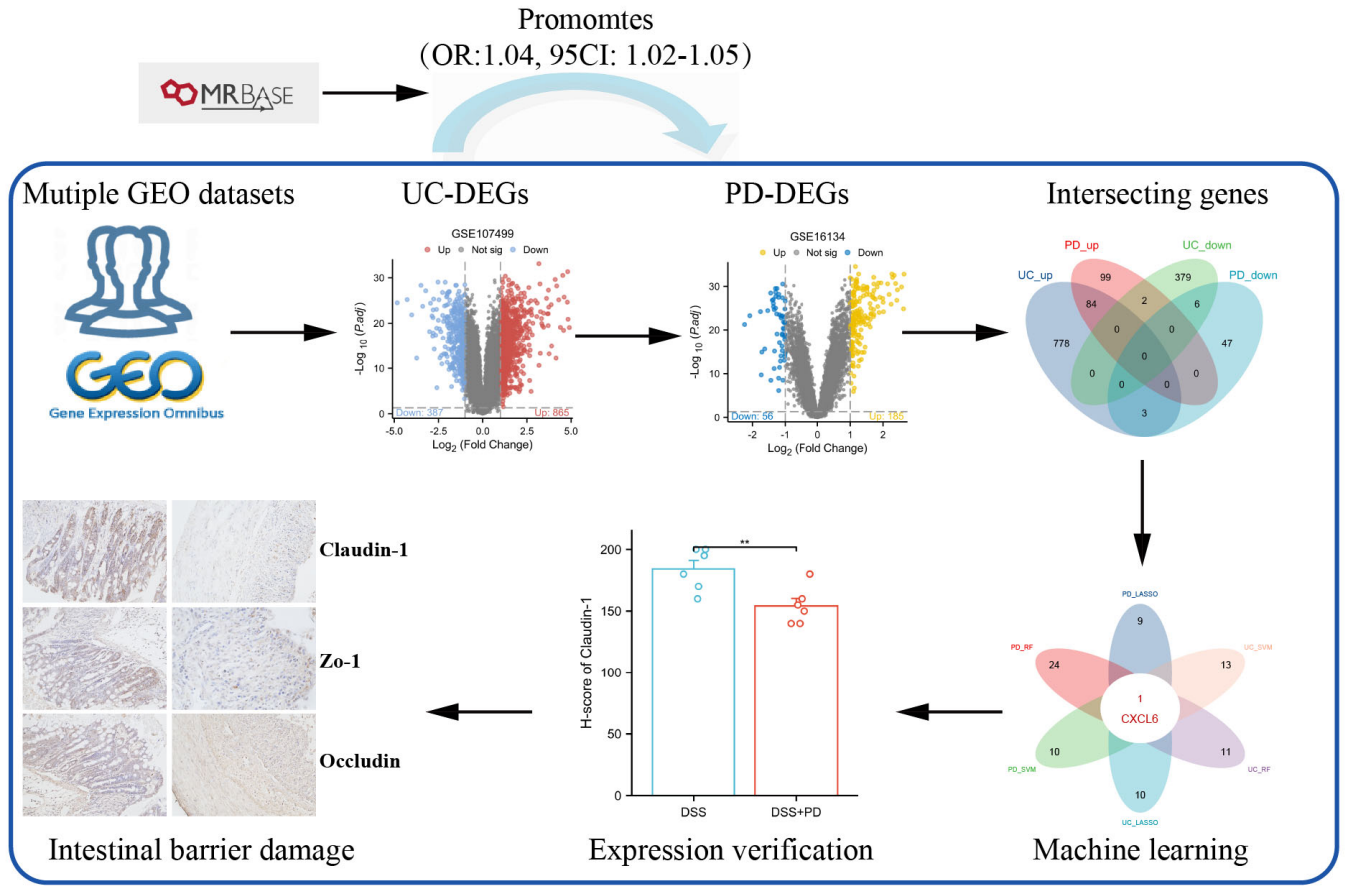
Flowchart.

The flowchart of the study is shown in **Figure 1**. Mendelian randomization (MR) analysis was performed using the MRbase online platform (https://app.mrbase.org) (21) to explore the causal relationship between UC and PD. UC data were sourced from several datasets available through the European Bioinformatics Institute (EBI), including ebi-a-GCST000964, ebi-a-GCST003045, ebi-a-GCST90018933, ebi-a-GCST90020072, as well as from the IEU OpenGWAS project, including ieu-a-32, ieu-a-968, ieu-a-970, and ieu-a-973. PD data were sourced from the finn-b-K11_PERIODON_CHRON dataset. All datasets are derived from European populations. None of the single nucleotide polymorphisms (SNPs) associated with PD met the threshold of P < 5 × 10^−8^; therefore, SNPs with P < 5 × 10^−6^ were selected as instrumental variables (IVs), following the criteria of previous studies (13, 22). For UC, SNPs with P < 5 × 10^−8^ were employed as IVs. Subsequently, MR analysis was performed on the website.

### 2.2 Meta-analysis

The combined odds ratios (OR) and 95% confidence intervals (CI) were calculated using R (version 3.6.0) with the Meta package (version 4.13-0). A fixed-effect model was applied to generate the forest plots. Data heterogeneity was assessed using the I² statistic, with I² ≥ 50% indicating high heterogeneity. Bias was evaluated using the Begg and Egger tests. A fixed-effect model was chosen based on low heterogeneity (I² < 50%) among studies.

### 2.3 Datasets collection and screening for differentially expressed genes

Microarray data for UC and PD were obtained from eight GEO databases, as detailed in **Supplementary Table S1**. All datasets underwent standardized preprocessing including background correction, log2 transformation, and quantile normalization using the “limma” package in R. Datasets selected for inclusion in this study were required to meet the following strict criteria: Adequate sample size: datasets with ≥10 samples per group (patients and controls). Clearly annotated clinical status: confirmed diagnoses of UC or PD and matched healthy controls. Transcriptomic profiles derived specifically from inflamed mucosal tissues (colonic tissues for UC and gingival tissues for PD). Availability of raw or properly normalized expression data suitable for integration and further analysis. Previous utilization in peer-reviewed immunological research studies to ensure biological relevance. Datasets were excluded if they had incomplete clinical annotation, insufficient sample size (<10 per group), data derived from non-mucosal tissues, or incompatible normalization status. For the analysis, data from GSE107499 and GSE16134 were preprocessed and normalized. Differentially expressed genes (DEGs) between disease and control groups were identified using the “limma” package in R, a standard tool for analyzing microarray data.

To visualize the DEGs, we used the “ggplot2” package to generate volcano plots and heatmaps. DEGs were considered statistically significant if they met the following criteria: adjusted p-value (adj. P) < 0.05 and absolute log2 fold change (|log2FC|) > 1. To identify common DEGs across the datasets, we intersected the DEGs from the two databases using a Venn diagram tool.

### 2.4 Functional enrichment analysis

To further investigate the biological significance of the shared DEGs identified from the datasets, functional enrichment analyses were performed. The analyses included Gene Ontology (GO), Kyoto Encyclopedia of Genes and Genomes (KEGG) pathway analysis, and Gene Set Enrichment Analysis (GSEA). All analyses were conducted using the “clusterProfiler” R package (23), and the “Hallmarks” pathway was selected for GSEA. To control for multiple hypothesis testing, false discovery rate (FDR) correction was applied using the Benjamini-Hochberg method in all enrichment analyses, including GO, KEGG, and GSEA. Enriched terms or pathways with an adjusted p-value (FDR) < 0.05 were considered statistically significant.

### 2.5 Screening of immune-related DEGs

To identify immune-related differentially expressed genes (IRDEGs), we obtained a comprehensive list of immune-related genes from the ImmPort database (https://www.immport.org/home), which provided a total of 2483 immune-related genes (IRGs). Then, we intersected the list of IRGs with the up-DEGs from UC and PD through Venn diagram tool.

### 2.6 Identification of optimal hub gene for UC and PD

We employed three machine learning methods: Random Forest (RF), Least Absolute Shrinkage and Selection Operator (LASSO), and Support Vector Machine Recursive Feature Elimination (SVM-RFE). For RF algorithm (24), we used the randomForest package in R with default parameters and set the number of trees to 500. The importance score of each gene was evaluated based on the mean decrease in accuracy and Gini index. For LASSO analysis, the glmnet package in R was utilized with cross-validation to determine the optimal regularization parameter (λ). Genes with non-zero coefficients were identified as key predictors (25). The e1071 package in R was used to perform SVM-RFE with a radial basis function (RBF) kernel. The number of features was sequentially reduced until the optimal number of features was determined (26). Hub genes identified by RF, LASSO, and SVM-RFE in both UC and PD datasets were intersected to find common genes across methods and conditions. Then, CXCL6 was highlighted as a critical gene due to its consistent identification across multiple machine learning approaches, suggesting its potential as a significant biomarker or therapeutic target in UC and PD.

### 2.7 Validation of CXCL6 expression

CXCL6 expression levels were validated in PD using GSE10334 and in UC using GSE47908. The sensitivity and specificity of CXCL6 as a diagnostic marker were evaluated using operating characteristic curves (ROC) and the area under the curve (AUC) by “pROC” package.

### 2.8 Subgroup classification of UC and PD patients based on CXCL6 expression

Based on the median expression value of CXCL6, UC and PD patients were categorized into different subgroups. DEGs between these subgroups were analyzed using the “limma” package, followed by Gene Set Enrichment Analysis (GSEA) (27) with Hallmark pathways. Additionally, immune infiltration and immune function across subgroups was assessed using single-sample GSEA (ssGSEA) using “GSVA” package, as previously mentioned (2).

### 2.9 The relationship between CXCL6 expression and biological therapy

Vedolizumab (VDZ) and Infliximab (IFX) are the most commonly used biologics for UC patients. We compared CXCL6 expression between responders and non-responders at baseline prior to biological therapy. The value of CXCL6 in predicting the efficacy of biological therapy was assessed using ROC curves and AUC. Additionally, we analyzed the changes in CXCL6 expression before and after treatment in responders.

### 2.10 Animals and experimental design

Male Sprague-Dawley rats (6–8 weeks old, weighing 200–250 g) were obtained from Beijing Huafukang Biotechnology Co., Ltd. (Beijing, China). All animals were housed in specific pathogen-free (SPF) conditions with a 12 h light/dark cycle and had ad libitum access to standard chow and water. The study protocol was approved by the Animal Ethics Committee of The Fifth Hospital of Shanxi Medical University (approval number: No. 361 of 2025).

To investigate the impact of periodontitis on the development of colitis, rats were randomly assigned to two experimental groups (n = 6 per group). In the DSS group, ulcerative colitis was induced by administering 3% dextran sulfate sodium (DSS)(MP Biomedicals, USA) in the drinking water for 7 consecutive days. In the UC + periodontitis group, rats underwent experimental periodontitis induction through repeated tooth abrasion. Specifically, the occlusal surfaces of the maxillary left molars were gently ground using a low-speed dental drill every 2–3 days for a total of 23 days. During the final 7 days of this period, the rats also received 3% DSS in their drinking water to induce colitis, concurrently modeling both periodontitis and UC.

### 2.11 Histological assessment (H&E staining)

Colon tissues were fixed in 4% paraformaldehyde, embedded in paraffin, sectioned at 5μm, and stained with hematoxylin and eosin (H&E). The scoring criteria included inflammatory cell infiltration, mucosal damage, glandular architecture distortion, and ulceration extent, each graded on a 0–4 scale, with a total maximum score of 16. Higher scores indicated more severe histopathological damage.

### 2.12 Immunohistochemistry

Paraffin-embedded colon tissues were sectioned at 5μm thickness for immunohistochemical analysis. After standard deparaffinization, rehydration, antigen retrieval, and blocking, sections were incubated overnight at 4°C with primary antibodies targeting Occludin, ZO-1, Claudin-1, and CXCL6 (all from Affinity Biosciences, China). The next day, slides were incubated with HRP-conjugated secondary antibody, followed by DAB development and hematoxylin counterstaining.

Stained sections were examined under a light microscope. Immunoreactivity was evaluated using the Hiscore semi-quantitative grading system, which assesses both the staining intensity and the proportion of positively stained cells across representative high-power fields.

### 2.13 Statistical analysis

All statistical analyses were conducted using R software (version 3.6.0). Two-sided tests were applied throughout the study. Wilcoxon rank-sum tests were employed for non-normally distributed data, while Student’s t-tests were used for normally distributed data. A p-value of less than 0.05 was deemed statistically significant.

## 3 Results

### 3.1 MR and meta-analysis

**Figure 2:**
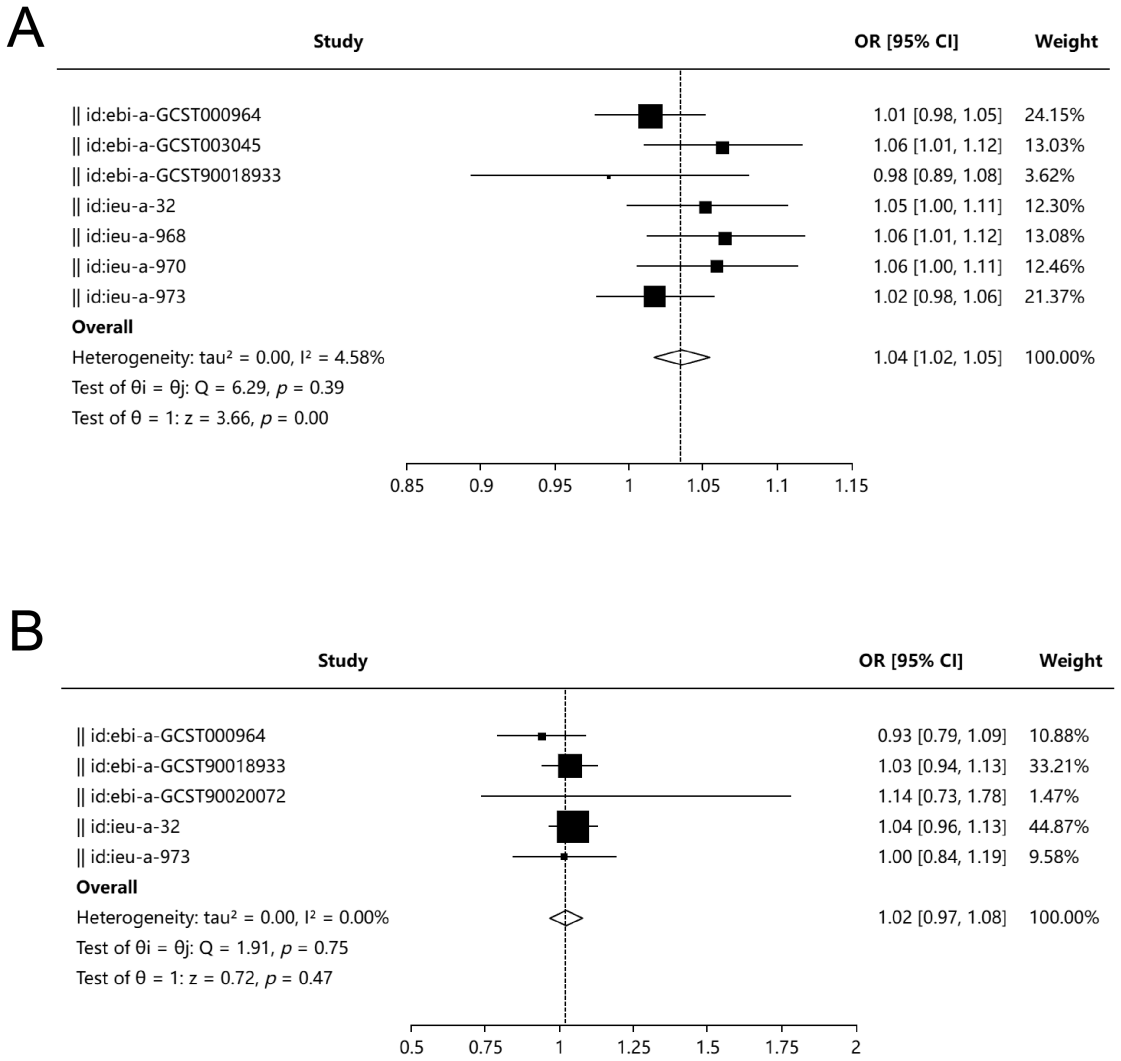
Bidirectional Mendelian randomization and meta-analysis between UC and PD. **(A)** Forest plot summarizing MR estimates from seven genome-wide association studies (GWAS) assessing the effect of UC on PD. Three datasets (ebi-a-GCST003045, ieu-a-968, ieu-a-970) demonstrated a significant association using the IVW method. Meta-analysis showed a significant causal effect of UC on PD (OR = 1.04; 95% CI, 1.02–1.05; P = 0.001; I² = 4.58%), with no evidence of publication bias as assessed by Egger’s test (P = 0.802) and Begg’s test (P = 0.881). **(B)** Forest plot of MR estimates evaluating PD as an exposure and UC as the outcome from five independent datasets. No significant causal association was observed (OR = 1.02; 95% CI, 0.97–1.08; P = 0.47; I² = 0%). Egger’s test (P = 0.724) and Begg’s test (P = 0.624) indicated no significant bias.

Among the eight UC databases used as exposures, MR analysis results were available for seven, with three databases (ebi-a-GCST003045, ieu-a-968, ieu-a-970) indicating that UC is a risk factor for PD, as assessed using the Inverse Variance Weighted (IVW) method. The meta-analysis demonstrated that UC is a risk factor for PD (OR, 1.04; 95% CI, 1.02–1.05; P = 0.001), with an I² of 4.58%. Both Egger’s test (P = 0.802) and Begg’s test (P = 0.881) revealed no evidence of bias (**Figure 2A**).

For UC as the outcome, MR analysis results were available from five databases, all of which showed that periodontitis is not a risk factor for UC. The meta-analysis yielded an OR of 1.02 (95% CI, 0.97–1.08; P = 0.47), with an I² of 0%. Neither Egger’s test (P = 0.724) nor Begg’s test (P = 0.624) indicated bias (**Figure 2B**).

### 3.2 Immune pathway associations of shared differentially expressed genes between UC and PD

**Figure 3:**
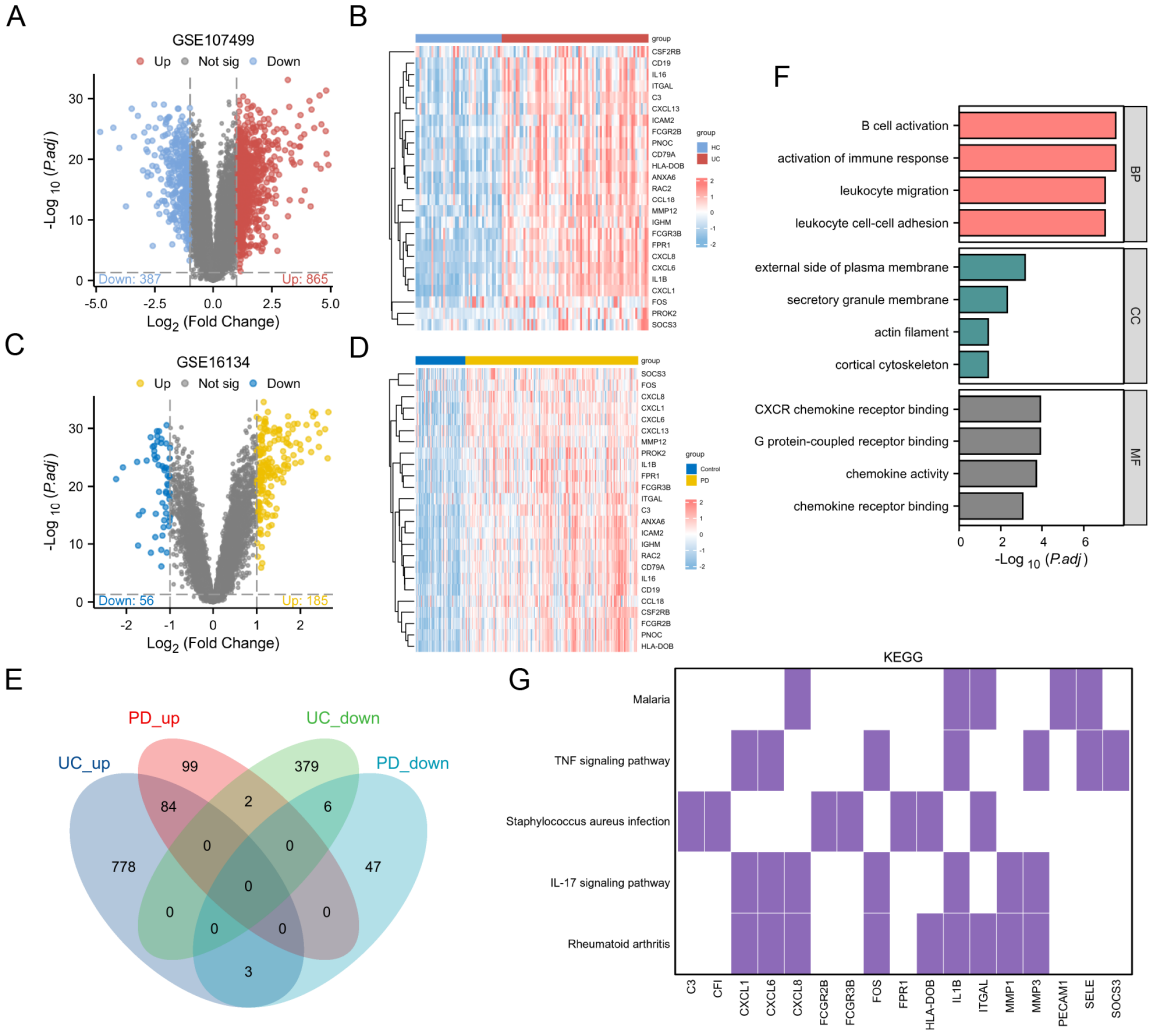
Differential Gene Expression and Functional Enrichment Analysis. **(A)** Volcano plot showing DEGs in the GSE107499 dataset. **(B)** Heatmap visualizing the expression profiles of DEGs across UC and healthy HC samples. **(C)** Volcano plot for the GSE16134 dataset (PD data). **(D)** Heatmap for the GSE16134 dataset, illustrating the expression profiles of DEGs across PD and control samples. **(E)** Venn diagram showing 84 upregulated and 6 downregulated genes common to both UC and PD conditions. **(F)** GO enrichment analysis results for the shared genes. **(G)** KEGG pathway analysis.

The differential gene expression analysis of the GSE107499 dataset identified 865 upregulated and 387 downregulated genes in UC (**Figure 3A**). A heatmap (**Figure 3B**) visualizes these expression profiles across UC and HC samples. For GSE16134 (PD data), the analysis revealed distinct upregulated and downregulated gene sets (**Figures 3C, D**).

A Venn diagram (**Figure 3E**) shows 84 upregulated and 6 downregulated genes common to both conditions, totaling 90 shared genes. These were subjected to functional enrichment analysis, revealing significant involvement in immune-related processes such as B cell activation and activation of immune response (**Figure 3F**). KEGG pathway analysis (**Figure 3G**) highlights the involvement of these genes in key immune pathways, including TNF signaling pathway and IL-17 signaling pathway, both crucial in UC and PD pathogenesis.

### 3.3 Machine learning approaches for identifying hub gene

**Figure 4:**
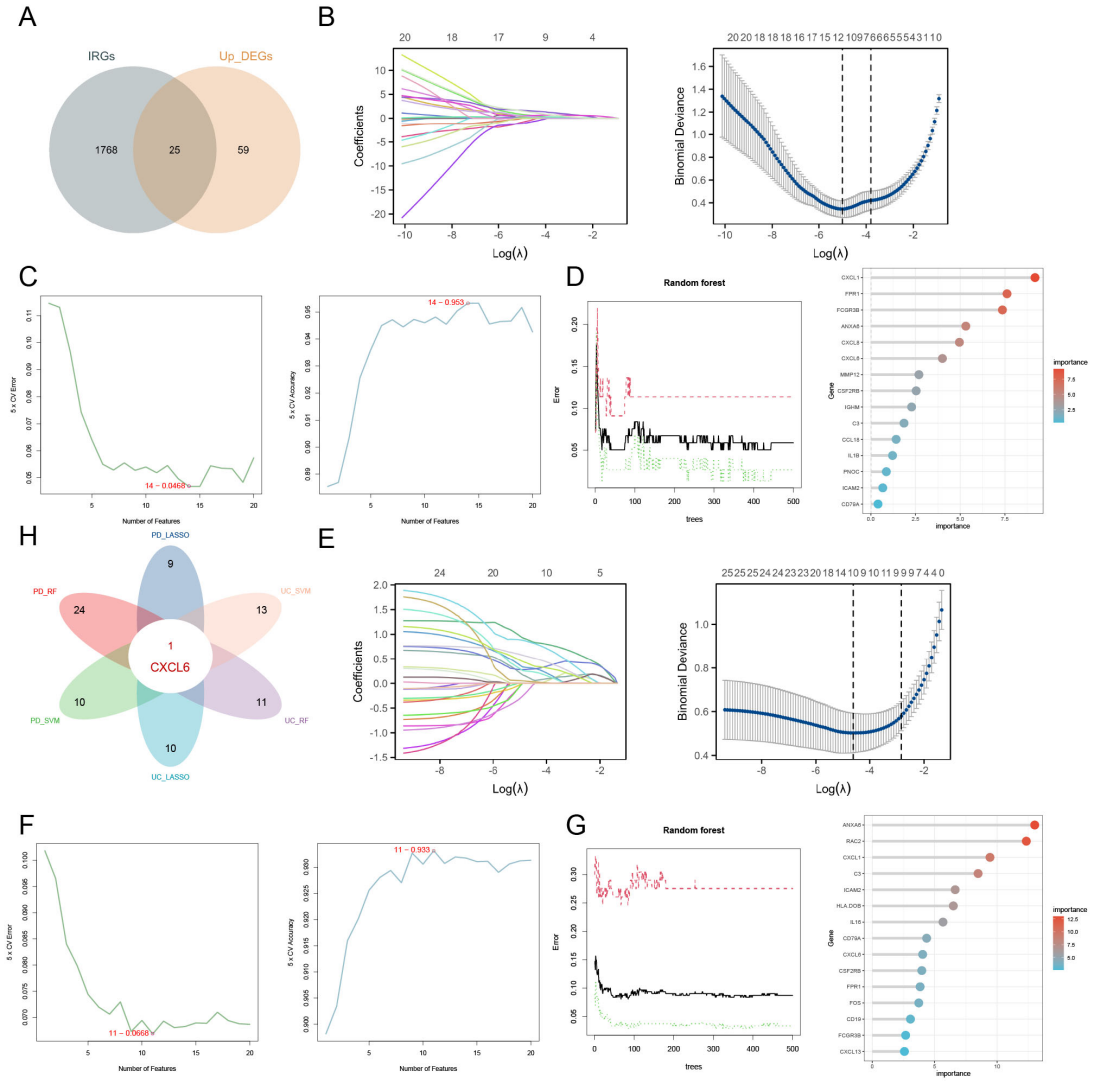
Identification of hub gene using machine learning approaches. **(A)** Venn diagram depicting the overlap between upregulated DEGs and IRGs. **(B)** LASSO analysis for UC identifying 11 genes. **(C)** SVM-RFE analysis selecting 14 genes. **(D)** RF analysis highlighting genes with an importance score greater than 1 as significant. **(E)** LASSO analysis for PD identifying 10 genes. **(F)** SVM-RFE analysis selecting 11 genes. **(G)** Random Forest results for PD. **(H)** Integration of machine learning results across both datasets pinpointing CXCL6 as the hub gene.

The Venn diagram illustrates the intersection between upregulated DEGs and immune-related genes (IRGs), identifying 25 candidate genes (**Figure 4A**). LASSO analysis of the GSE107499 dataset (UC data) identified 11 genes (**Figure 4B**). SVM-RFE analysis selected 14 genes with notable accuracy (0.953) and a minimal error rate (0.0468) (**Figure 4C**). For RF analysis, genes with an importance score greater than 1 are considered significant (**Figure 4D**). For the GSE16134 dataset (PD data), LASSO analysis identified 10 genes (**Figure 4E**), while SVM-RFE analysis selected 11 genes with an accuracy of 0.933 and a minimal error rate of 0.0668 (**Figure 4F**). Random Forest results for PD are shown in (**Figure 4G**). The intersection of the results from these machine learning methods across both datasets led to the identification of CXCL6 as the hub gene (**Figure 4H**).

### 3.4 Model evaluation

**Figure 5:**
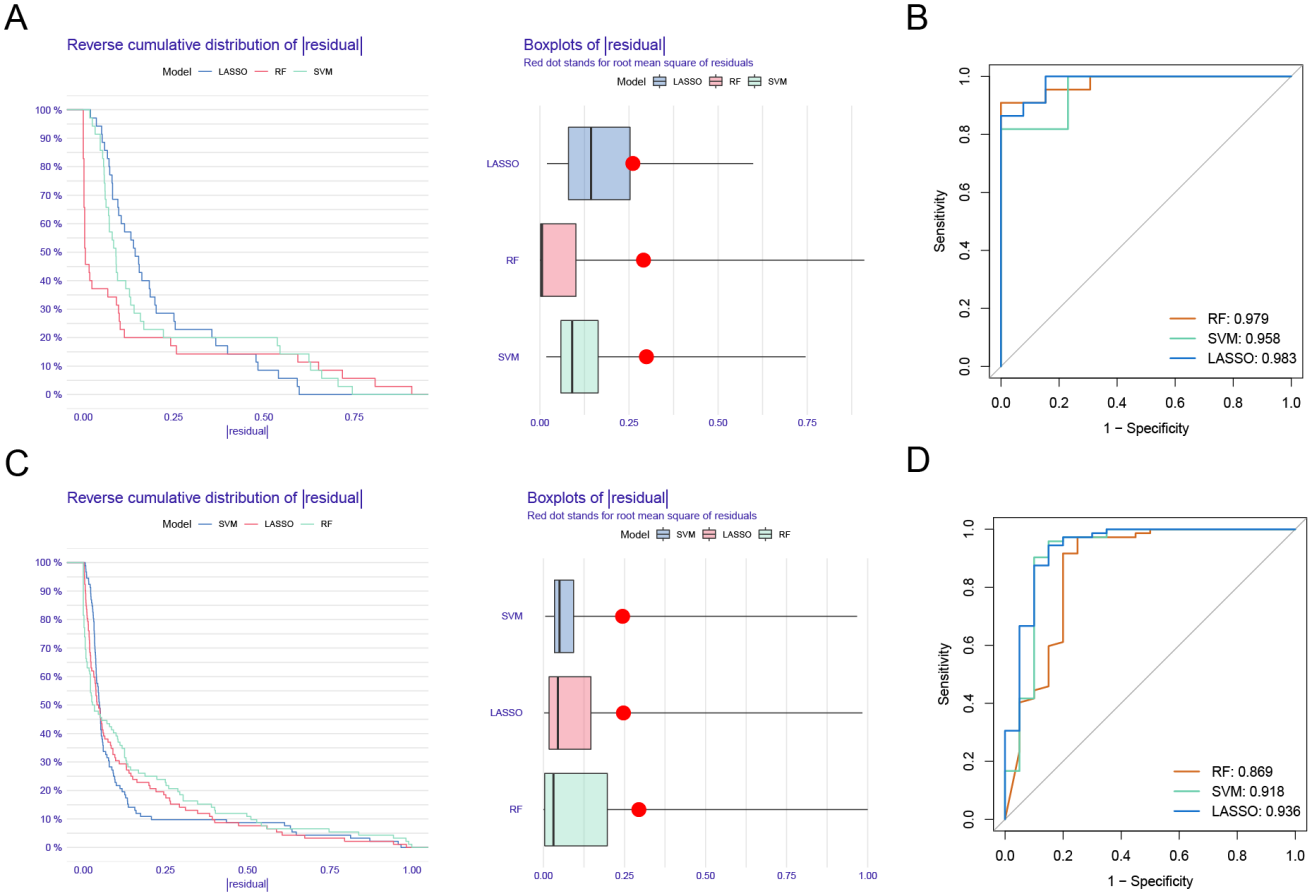
Model verification. **(A)** Reverse cumulative distribution plot and boxplots showing residual expression patterns of machine learning models in UC. **(B)** Diagnostic performance of RF, SVM, and LASSO. **(C)** Residual expression patterns for machine learning models in PD are shown. **(D)** The models demonstrating significant diagnostic value for PD.

The reverse cumulative distribution plot and boxplots illustrate the residual expression patterns of machine learning models in UC, highlighting model variations (**Figure 5A**). Diagnostic performance metrics show high accuracy for UC, with AUC values of 0.979 for RF, 0.958 for SVM, and 0.983 for LASSO (**Figure 5B**). Similarly, residual expression patterns for machine learning models in PD are shown (**Figure 5C**), with the models demonstrating significant diagnostic value for PD (**Figure 5D**).

### 3.5 Validation of CXCL6 expression and diagnostic value

**Figure 6:**
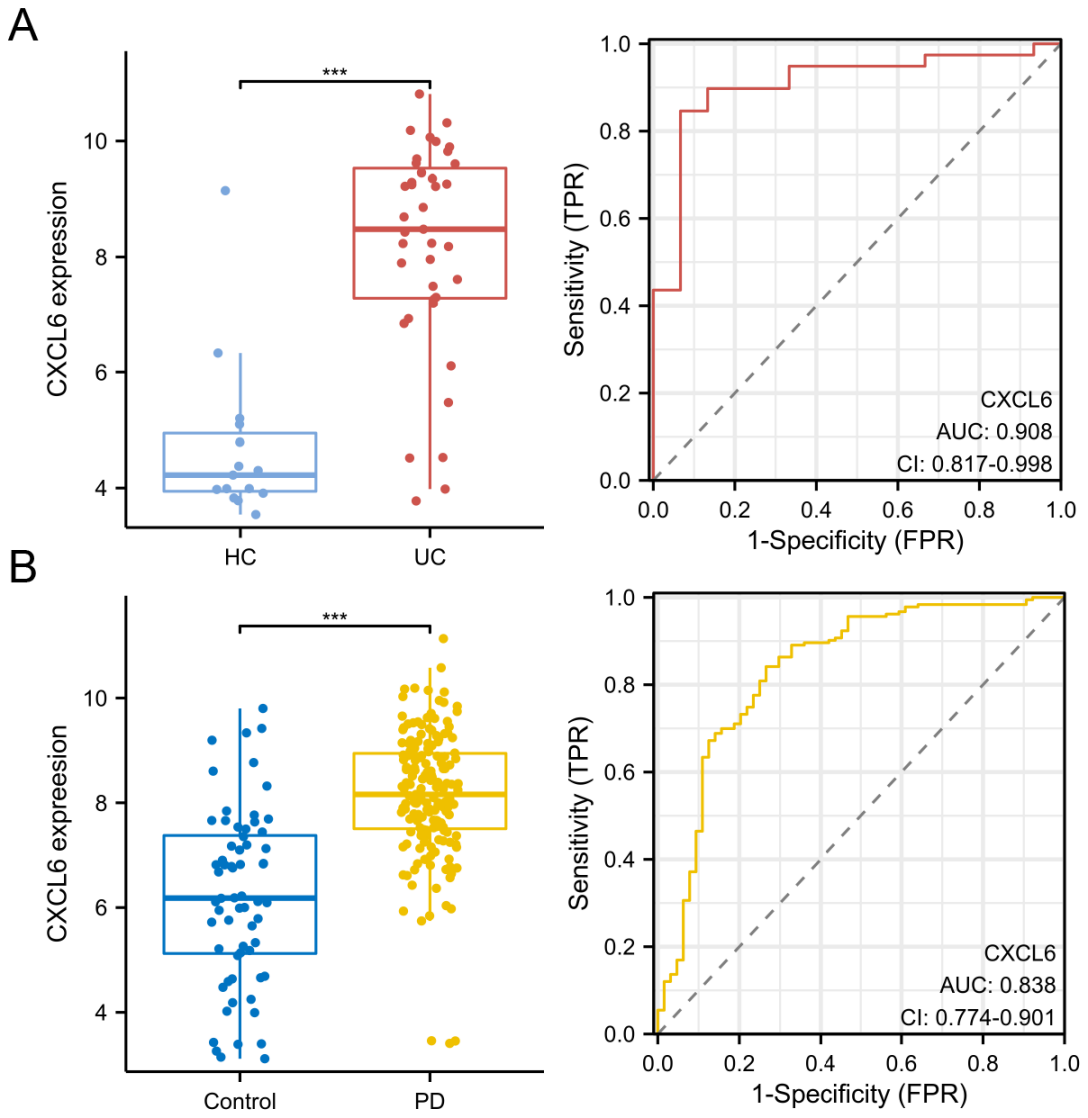
Validation of CXCL6 expression and diagnostic value. **(A)** Expression of CXCL6 in disease and control groups, validated using the GSE47908 dataset. **(B)** Expression of CXCL6 in disease and control groups, validated using the GSE10334 dataset. ROC curves demonstrate the diagnostic value of CXCL6. Results show that CXCL6 expression is significantly upregulated in both UC and PD samples and exhibits strong diagnostic potential. ***P<0.001.

We further validated CXCL6 expression in disease and control groups using the GSE47908 dataset (UC data) and the GSE10334 dataset (PD data). ROC curve analysis demonstrated the diagnostic potential of CXCL6. The results indicate that CXCL6 is significantly upregulated in both UC (**Figure 6A**) and PD patients (**Figure 6B**) and exhibits strong diagnostic value.

### 3.6 Immune pathway activation and immune cell enrichment in CXCL6 high-expression patients

**Figure 7:**
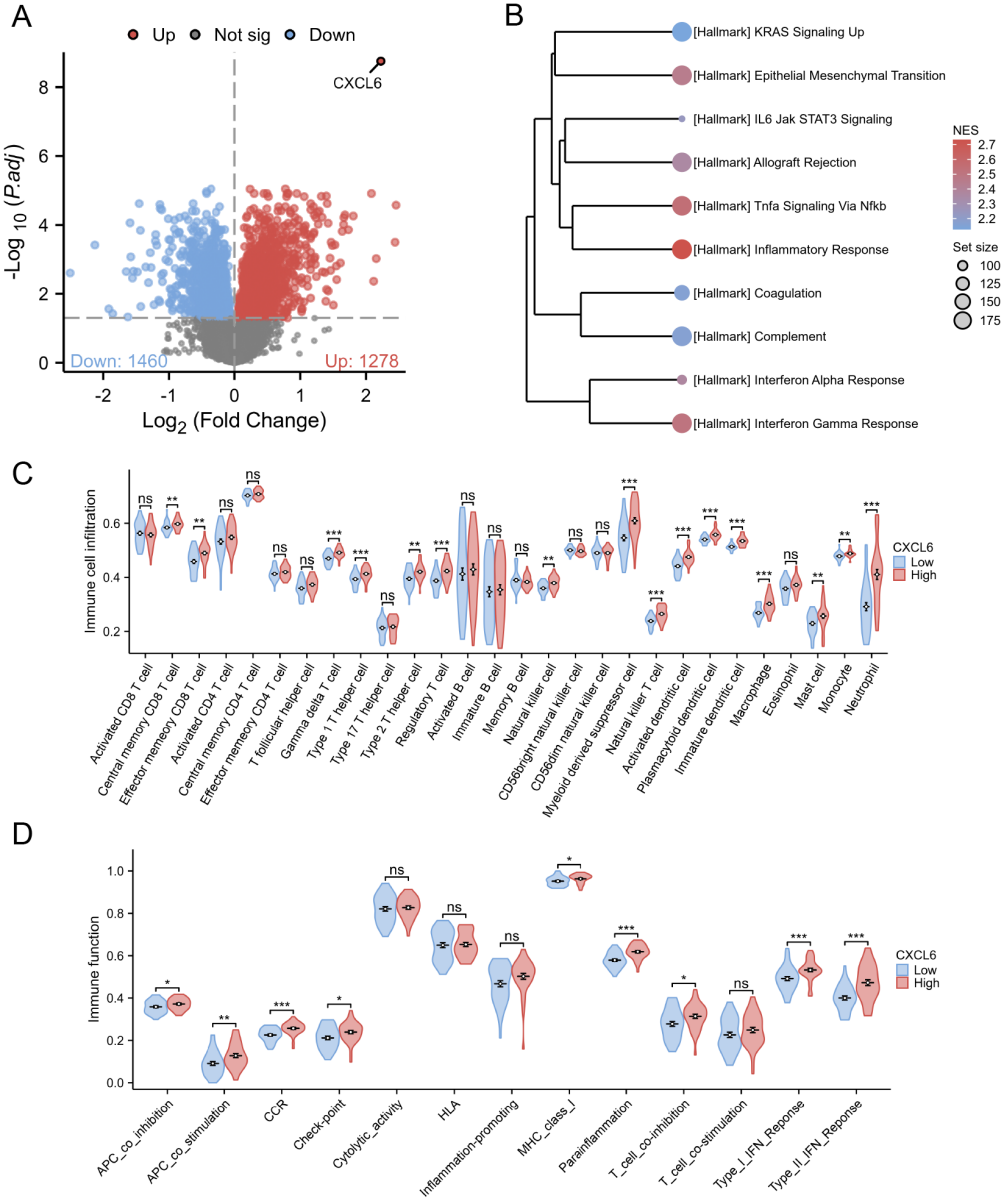
Subgroup analysis based on CXCL6 expression in UC patients. **(A)** Differential gene analysis between CXCL6 high-expression and low-expression groups based on the median CXCL6 expression in the GSE107499 dataset. **(B)** GSEA analysis about Hallmark pathways. **(C)** Immune infiltration analysis indicating increased levels of immune cell in CXCL6 high-expression group. **(D)** Enrichment of various immune-related pathways in the CXCL6 high-expression group. *P<0.05, **P<0.01, ***P<0.001. ns, not significant.

We divided UC patients from the GSE107499 dataset into CXCL6 high-expression and low-expression groups based on the median expression of CXCL6. Differential gene analysis between these groups is shown (**Figure 7A**). GSEA using Hallmark pathways revealed that the CXCL6 high-expression group had higher activation levels in immune pathways, such as TNF signaling via NF-kB and inflammatory response, compared to the low-expression group (**Figure 7B**). Immune infiltration analysis indicated a significant increase in immune cells involved in UC development, such as macrophages and neutrophils, in the CXCL6 high-expression group (**Figure 7C**). Similarly, various immune-related pathways, including APC stimulation and IFN response, were enriched in the CXCL6 high-expression group (**Figure 7D**).

**Figure 8:**
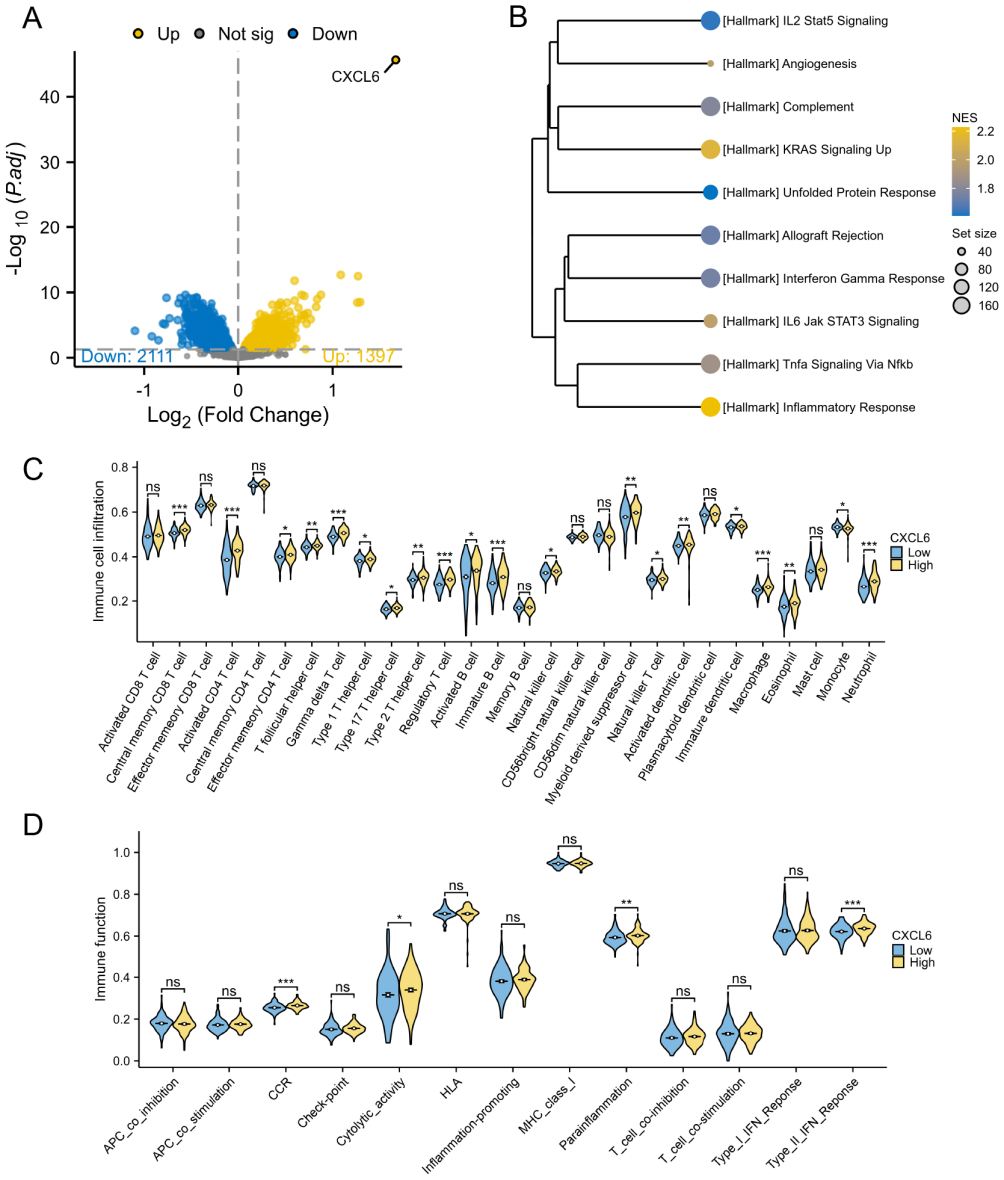
Subgroup analysis based on CXCL6 expression in PD patients. **(A)** Differential gene analysis between CXCL6 high-expression and low-expression groups. **(B)** GSEA analysis indicating enrichment of immune pathways in the CXCL6 high-expression group. **(C)** Immune cell infiltration analysis showing increased levels of immune cells in the CXCL6 high-expression group. **(D)** Immune function scoring highlighting significant immune activity in the CXCL6 high-expression group. *P<0.05, **P<0.01, ***P<0.001. ns, not significant.

In a comparable manner, the PD cohort from the GSE16134 dataset was analyzed for CXCL6 high-expression and low-expression groups, revealing 1397 upregulated and 2111 downregulated genes (**Figure 8A**). GSEA showed that the CXCL6 high-expression group exhibited significant enrichment in immune pathways (**Figure 8B**). Immune cell infiltration analysis also indicated a notable increase in immune cell levels in the CXCL6 high-expression group (**Figure 8C**). Additionally, immune function scoring demonstrated elevated immune activity in the CXCL6 high-expression group (**Figure 8D**).

### 3.7 The relationship between CXCL6 expression and biological therapy

**Figure 9:**
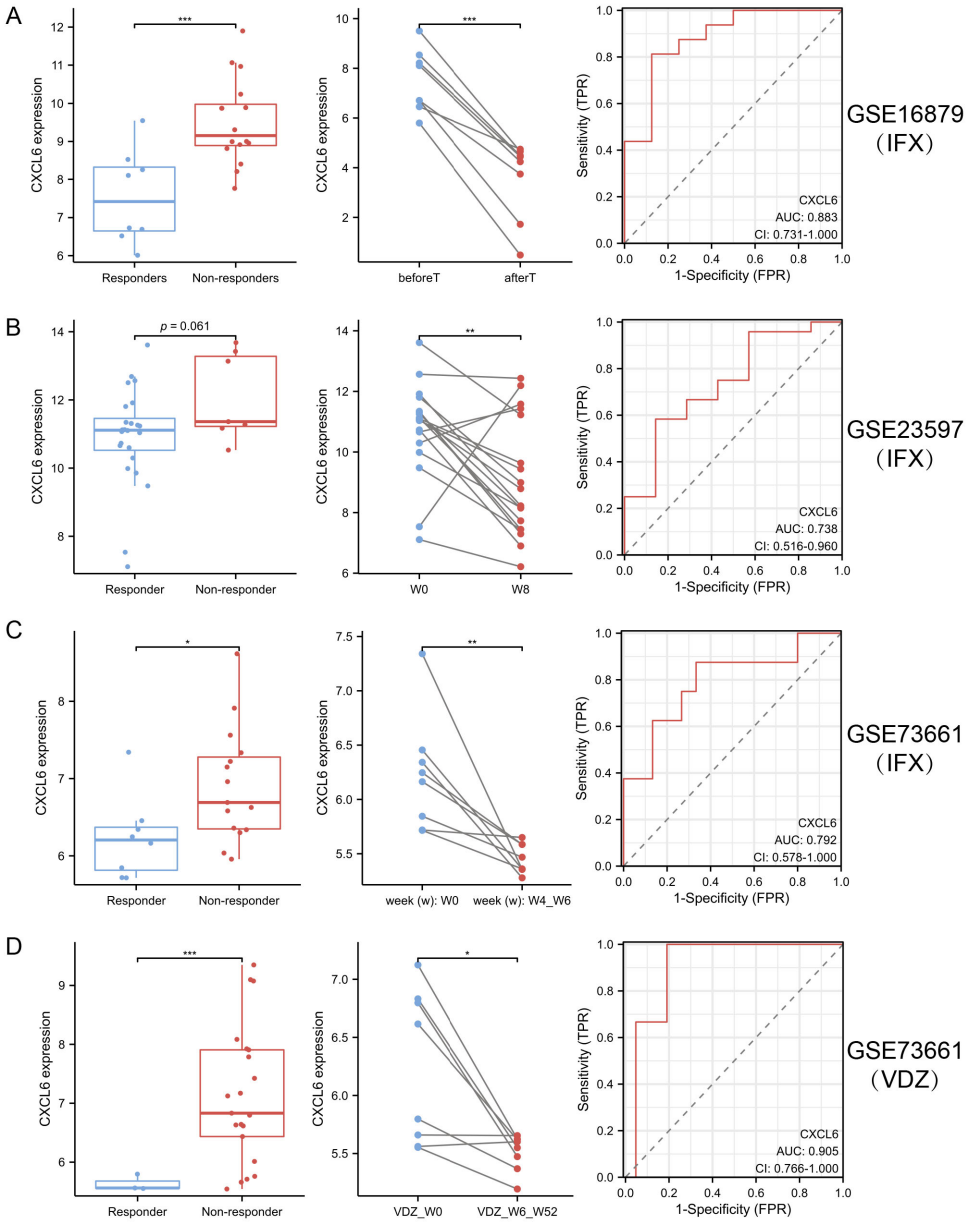
CXCL6 expression predicts response to biologic therapy in ulcerative UC. **(A–D)** CXCL6 expression was analyzed in publicly available transcriptomic datasets from UC patients receiving biologic therapy: GSE16879 **(A)**, GSE23597 **(B)**, and GSE73661 before and after IFX **(C)** or VDZ **(D)** treatment. In both cohorts, baseline CXCL6 expression was significantly higher in non-responders compared to responders, suggesting its potential as a predictive biomarker. In responders, CXCL6 expression was markedly reduced following IFX or VDZ therapy. *P<0.05, **P<0.01, ***P<0.001.

In the analysis of GSE16879 (**Figure 9A**), GSE23597 (**Figure 9B**), GSE73661 (**Figure 9C**), and GSE73661 (**Figure 9D**), CXCL6 expression was assessed to evaluate its potential as a predictive biomarker for biologic treatment efficacy, particularly for VDZ (AUC > 0.9). The results revealed that patients with elevated CXCL6 levels, indicating a higher inflammatory burden, were more likely to be non-responders to biologics. Furthermore, in responders, both IFX and VDZ treatments significantly reduced CXCL6 expression. These findings highlight the potential benefit of biologics for UC patients with periodontitis, as CXCL6 serves as a common hub gene linking UC and PD. It is essential to recognize that the oral-gut axis suggests that managing intestinal inflammation may also play a role in mitigating oral diseases. Consequently, therapies such as VDZ, which primarily focus on the gastrointestinal tract, may inadvertently benefit periodontitis related to ulcerative colitis.

### 3.8 DSS+PD treatment exacerbates colitis with elevated CXCL6 expression and barrier dysfunction

**Figure 10:**
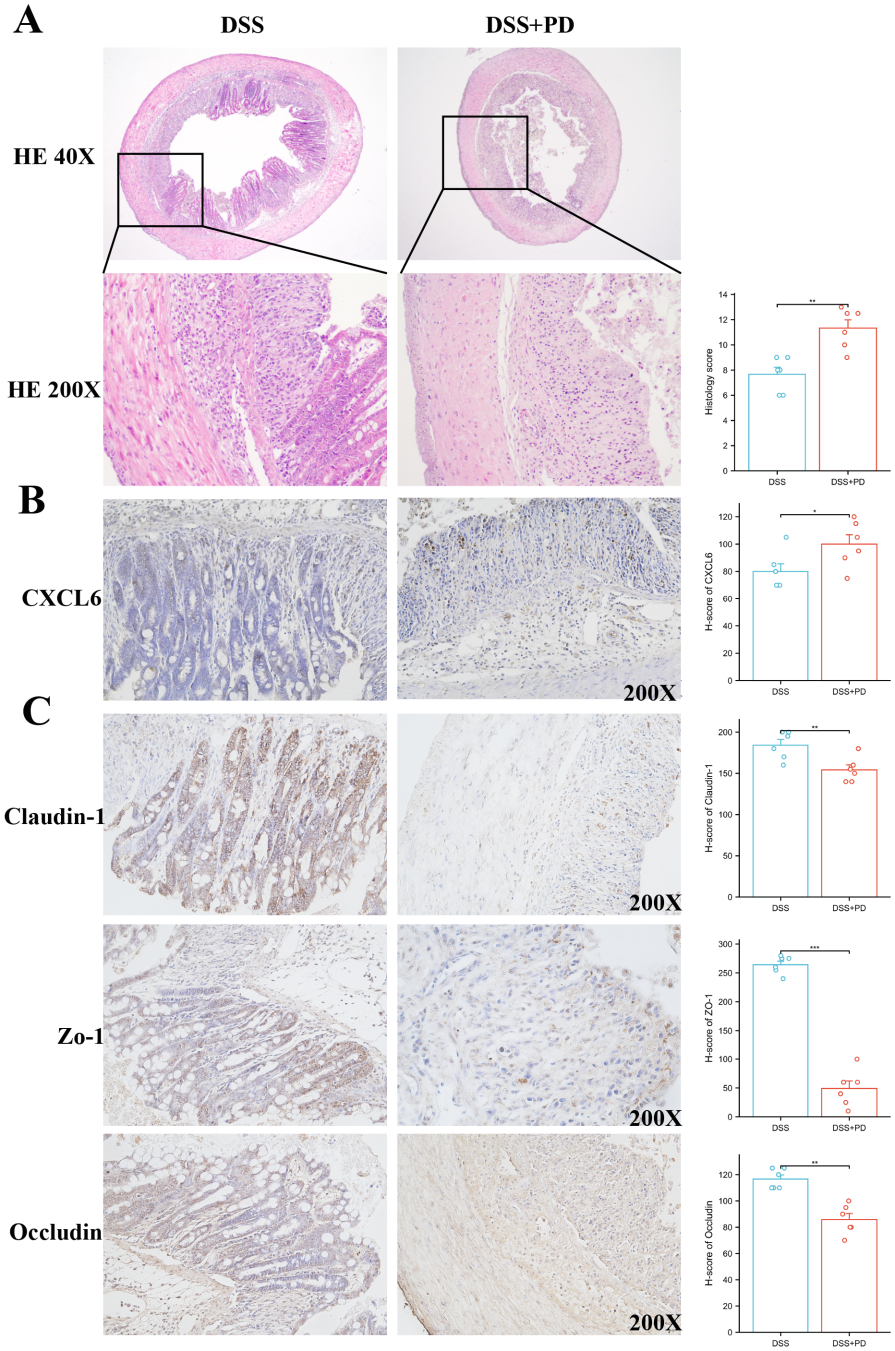
Combined DSS and PD treatment enhances colonic inflammation, increases CXCL6 expression, and impairs epithelial barrier integrity. **(A)** Representative H&E staining of colon sections from DSS and DSS+PD-treated. The DSS+PD group exhibited increased inflammatory cell infiltration and epithelial damage compared to DSS alone. Histological scores (right panel) reflect significantly aggravated colitis in the DSS+PD group. **(B)** Immunohistochemical staining for CXCL6 in colonic tissues showed higher expression levels in the DSS+PD group compared to DSS alone. Quantification by H-score confirmed significantly elevated CXCL6 expression. **(C)** Immunostaining of tight junction proteins Claudin-1, ZO-1, and Occludin revealed reduced expression in the DSS+PD group, indicating impaired intestinal epithelial barrier function. H-score analysis showed significant downregulation of all three markers. *P<0.05, **P<0.01, ***P<0.001.

To validate our bioinformatic prediction of CXCL6 upregulation under compound disease conditions, we established a rat model of colitis induced by DSS in combination with PD treatment. Histological examination revealed markedly heightened immune cell infiltration and epithelial disruption in the DSS+PD group relative to DSS alone (**Figure 10A**). CXCL6 expression, assessed by immunohistochemistry, was significantly elevated in colonic tissues from the DSS+PD group (**Figure 10B**). Given the enhanced inflammatory response, we next evaluated intestinal barrier integrity by staining for the tight junction proteins Claudin-1, ZO-1, and Occludin. All three markers were reduced in the DSS+PD group, indicating compromised epithelial barrier function (**Figure 10C**). These findings confirm that CXCL6 is upregulated under dual inflammatory challenge and coincides with more severe epithelial damage. While the mechanistic link remains to be established, the parallel increase in CXCL6 expression and loss of barrier proteins raises the possibility that CXCL6 may contribute to intestinal barrier dysfunction in this context.

## 4 Discussion

The bidirectional relationship between oral diseases and UC has gained considerable attention in recent research. Mechanistically, UC may immunologically influence oral health through the recognition of shared epitopes across different body sites. Extraintestinal manifestations of UC are thought to arise from a systemic adaptive immune response, which is initiated by local dysbiosis in the gut (28). On the other hand, the co-occurrence of oral dysbiosis, which promotes the proliferation of oral pathogens, and impaired gut mucosal immunity, characterized by a compromised intestinal barrier against oral microbes, creates a permissive environment for the colonization of the gut by oral pathogens. This microbial invasion exacerbates intestinal inflammation by triggering both innate and adaptive immune responses (29).

We conducted bidirectional MR analysis to examine the causal relationship between UC and PD. Our findings indicate that UC increases the risk of PD, but not vice versa. Interestingly, to date, three MR have examined the relationship between UC and PD, yet they have reported conflicting results (13, 14, 22). Notably, our study aligns with the findings of Wang et al. (14). One possible explanation for the discrepancies in previous studies may be the selection of different GWAS databases. Our study, however, benefits from a meta-analysis of multiple GWAS datasets, which strengthens the robustness of our conclusions. Interestingly, the lack of reverse causality (PD to UC) may reflect asymmetry in systemic immune activation, or limitations in the power of PD GWAS datasets. It is also possible that UC, as a systemic immune disease, exerts broader downstream effects compared to localized PD.

Understanding the shared mechanisms between UC and PD could provide critical insights into addressing PD associated with UC. Using multiple GEO databases, we identified DEGs common to both UC and PD, many of which are linked to the activation of various immune pathways. We intersected DEGs with immune-related genes and applied three machine learning algorithms, identifying CXCL6 as a shared immune mediator. Validation in independent GEO datasets further supports CXCL6’s robustness as a potential biomarker. Based on the expression levels of CXCL6, we further stratified the UC and PD cohorts into distinct subgroups. The results consistently revealed that patients with high CXCL6 expression exhibited increased activation of immune pathways and enrichment of immune cells. This finding highlights the potential role of CXCL6 in modulating immune responses in both UC and PD, suggesting its relevance as a biomarker for immune dysregulation in these conditions. CXCL6 was identified as a shared immune hub gene between UC and PD, suggesting its potential role in mucosal inflammation and barrier dysfunction. Our enrichment analysis demonstrated that CXCL6 is closely associated with IL-17 and TNF signaling pathways, both of which are major drivers of neutrophil recruitment and mucosal inflammation in UC and PD. CXCL6 may act upstream, enhancing neutrophil chemotaxis via CXCR2 and thereby reinforcing IL-17/TNF-mediated inflammatory loops and barrier dysfunction.

Emerging evidence suggests that the treatment of UC may positively influence the outcomes of PD, supporting the hypothesis that both conditions share common immunoinflammatory pathways. UC patients undergoing anti-TNF biologic therapy have shown notable improvement in apical periodontitis, with faster healing compared to untreated controls (30). Additionally, in UC patients responsive to biologic therapy, significant increases in salivary levels of IgA and MPO have been observed, indicating that effective UC treatment may also enhance oral immune defense mechanisms (31). Furthermore, biologic agents used for other chronic inflammatory diseases have demonstrated the ability to slow the progression of PD and promote healing following periodontal therapy (32). Given these findings, we examined the relationship between CXCL6, the hub gene identified in our study, and biologic treatment. Our results demonstrate that elevated CXCL6 expression correlates significantly with non-response to biologic therapies commonly used in ulcerative colitis (UC), such as Vedolizumab and Infliximab. These findings suggest CXCL6 could serve as a valuable predictive biomarker, aiding clinicians in identifying patients less likely to respond to specific biologics, thus optimizing therapeutic strategies. While our retrospective analysis suggests that CXCL6 may predict response to biologic therapy in UC patients, it is important to acknowledge potential confounding by oral inflammation due to concomitant PD. Elevated CXCL6 levels in UC patients with PD might reflect oral inflammatory burden rather than intestinal-specific disease activity, which could limit its predictive specificity.

Our results experimentally validate elevated CXCL6 expression under combined inflammatory conditions. In a murine model of colitis induced by DSS and PD co-treatment, we observed a significant increase in CXCL6 expression that paralleled exacerbated tissue inflammation and barrier dysfunction. These observations are consistent with previous studies implicating CXCL6 in neutrophil recruitment and inflammatory amplification (33, 34). This may be mechanistically explained by CXCL6-induced neutrophil recruitment via CXCR2 activation and downstream NF-κB signaling, which disrupts tight junction protein expression and amplifies mucosal inflammation.

CXCL6 upregulation coincided with reduced expression of tight junction proteins (Claudin-1, ZO-1, Occludin). Although causality remains unproven, this correlation suggests CXCL6 may disrupt epithelial barrier integrity during chronic inflammation. Given the central role of the epithelial barrier in maintaining intestinal homeostasis, further investigation into the functional role of CXCL6 in barrier regulation will be of interest.

This study has several limitations. First, regarding our MR analysis, due to the lack of genome-wide significant SNPs (P < 5 × 10^-8^) for PD, we adopted a relaxed significance threshold (P < 5 × 10^-6^) to select instrumental variables. While this approach is consistent with previous studies, it may reduce the strength and reliability of causal inference by potentially introducing weak instruments or increasing pleiotropy. Sensitivity analyses and future MR studies using larger GWAS datasets will be necessary to strengthen causal inference. Second, although our findings convincingly demonstrate an association between CXCL6 expression and aggravated mucosal inflammation and barrier dysfunction in UC and PD, direct functional validation is still lacking. Our ongoing experiments using CXCL6-neutralizing antibodies and planned studies involving gene knockdown or knockout models will be essential to confirm its mechanistic role. Third, all GWAS and transcriptomic datasets analyzed in this study were derived from European populations. Given that genetic susceptibility, immune responses, and disease phenotypes can differ across ethnic groups, future studies should validate our findings in more diverse populations, including Asian and African cohorts, to ensure broader applicability. Finally, our rat periodontitis model relied on mechanical tooth abrasion, which, while reproducible and controlled, does not fully mimic the microbial dysbiosis that characterizes human PD. Future research should incorporate microbial analyses (e.g., 16S rRNA sequencing) and consider alternative models such as ligature-induced or bacterial inoculation models to better reflect human disease pathophysiology.

Collectively, these results suggest that CXCL6 is not only a marker of aggravated colitis under dual stress but may also play a mechanistic role in disease progression. Future studies using CXCL6-deficient models or targeted inhibition strategies will be necessary to delineate its contribution to epithelial damage and inflammation.

## Data Availability

The original contributions presented in the study are included in the article/**Supplementary Material**. Further inquiries can be directed to the corresponding authors.
