# Supplementary material for: Shared immune-inflammatory mechanisms between ulcerative colitis and periodontitis: a multi-omics analysis
Source: Front Immunol. 2025 Aug 27;16:1668277. doi: 10.3389/fimmu.2025.1668277 (PMC12420251; doi:10.3389/fimmu.2025.1668277)
Supplement: Supplementary file 1 [file Table1.docx]

Table 1. The information of all GEO cohorts.

| Dataset | Platform | Samples | Disease | Group |
| --- | --- | --- | --- | --- |
| GSE16134 | GPL570 | 241 cases and 69 controls | PD | Discovery |
| GSE107499 | GPL15207 | 75 cases and 44 controls | UC | Discovery |
| GSE10334 | GPL570 | 183 cases and 64 controls | PD | Validation |
| GSE47908 | GPL570 | 39 cases and 15 controls | UC | Validation |
| GSE73661 | GPL6244 | 47 cases | UC | Infliximab  (R: 8; NR: 15)  Vedolizumab  (W6_R: 3;W52_R:8) |
| GSE16879 | GPL570 | 24 cases | UC | Infliximab  (R: 8; NR: 16) |
| GSE23597 | GPL570 | 31 cases | UC | Infliximab  (W8_R: 24; NR: 7) |
